# Supplementary material for: Cross-Sectional Association between Perceived Physical Literacy and Mediterranean Dietary Patterns in Adolescents: The EHDLA Study
Source: Nutrients. 2023 Oct 17;15(20):4400. doi: 10.3390/nu15204400 (PMC10610224; doi:10.3390/nu15204400)
Supplement: Supplementary file 1 [file nutrients-15-04400-s001.zip › nutrients-2617235-SI.pdf]

Table S1. Mediterranean Diet Quality Index in children and adolescents according to perceived physical literacy status.

KIDMED items	Low PPL (9–31 Points)	Medium PPL (32–36 Points)	High PPL (31–45 Points)	*p*
Fruit or fruit juice daily	196 (67.1)	231 (76.0)	200 (80.3)	0.001
Second serving of fruit daily	88 (30.1)	127 (41.8)	119 (47.8)	<0.001
Fresh or cooked vegetables daily	188 (64.4)	218 (71.7)	195 (78.3)	0.002
Fresh or cooked vegetables >1/day	78 (26.7)	115 (37.8)	91 (36.5)	0.008
Regular fish consumption (at least 2-3/week)	149(51)	183(60.2)	174(69.9)	<0.001
>1/week fast-food (hamburger) restaurant	97(33.2)	95(31.2)	65(26.1)	0.185
Pulses >1/week	209(71.6)	244(80.3)	198(79.5)	0.023
Pasta or rice almost daily (≥5 days/week)	174 (59.6)	162 (53.3)	144 (57.8)	0.278
Cereal or cereal product for breakfast	155 (53.1)	188 (61.8)	156 (62.7)	0.037
Regular nut consumption (at least 2-3/week)	156(53.4)	176(57.9)	162(65.1)	0.023
Use of olive oil at home	275(94.2)	302(99.3)	245(98.4)	<0.001
No breakfast	56(19.2)	44(14.5)	27(10.8)	0.024
Dairy product for breakfast	206(70,5)	237(78,0)	202(81,1)	0.011
Commercially baked goods or pastries for breakfast	58(19,9)	46(15,1)	29(11,6)	0.031
Two yoghurts and/or 40g cheese daily	96(32,9)	121(39,8)	103(41,4)	0.088
Sweets and candy several times a day	76 (26.0)	64 (21.1)	51 (20.5)	0.221

KIDMED, Mediterranean Diet Quality Index in children and adolescents; PPL, perceived physical literacy.
